# Lipid metabolism dysregulation in Parkinson’s disease: A Mendelian randomization and transcriptomic analysis

**DOI:** 10.1016/j.ibneur.2026.08.012

**Published:** 2026-08-26

**Authors:** Fatma Zehra Calikusu, Suxiang Chen, Tao Wang, Dunhui Li

**Affiliations:** a Stephen & Denise Adams Center for Parkinson's Disease Research, Yale School of Medicine, New Haven, CT 06510, USA; b Department of Neurology, Yale School of Medicine, New Haven, CT 06510, USA; c Personalized Medicine Center, Health Futures Institute, Murdoch University, Murdoch, WA 6150, Australia; d The Kids Research Institute Australia, The University of Western Australia, Nedlands, WA 6009, Australia

**Keywords:** Parkinson's disease, Lipid metabolism, Mendelian randomization, Biomarkers

## Abstract

**Background:**

Parkinson’s disease (PD) is a progressive neurodegenerative disorder in which mechanisms linking metabolic dysregulation to neuronal vulnerability remain incompletely understood. Increasing evidence suggests that dysregulation of lipid metabolism plays an important role in modulating mitochondrial function, protein homeostasis, and neuroinflammation. In this study, we aimed to identify lipid metabolism-associated molecular drivers of PD and explore their potential contribution to the disease.

**Methods:**

Gene expression datasets GSE20141 and GSE20292 were obtained from the Gene Expression Omnibus (GEO) public database. Weighted Gene Co-expression Network Analysis (WGCNA) was performed to identify PD-associated modules, which were intersected with differentially expressed genes and curated lipid metabolism gene sets to define candidate lipid metabolism-related genes (LMRGs). Two machine learning algorithms, LASSO regression and Boruta, were applied to identify potential biomarkers. Diagnostic performance was evaluated using receiver operating characteristic (ROC) analysis. Gene Set Enrichment Analysis (GSEA), immune infiltration analysis, and Mendelian randomization (MR) were performed to assess biological functions and causality. Drug–gene interactions were predicted using the DGIdb.

**Results:**

Three genes: DGKD, LEP, and GGPS1, were consistently identified as candidate PD-associated LMRGs with reproducible diagnostic performance (AUC > 0.7). Among these, GGPS1, a key enzyme in the mevalonate pathway regulating protein prenylation, showed enrichment in the neurotrophin signaling pathway and was associated with central memory T cells. These findings suggest a mechanistic link between lipid metabolism, small GTPase prenylation, and neuronal homeostasis. Drug-gene interaction analysis identified compounds targeting GGPS1, including bisphosphonate derivatives, as potential therapeutic candidates for further investigation.

**Conclusion:**

This integrative analysis identified DGKD, LEP, and GGPS1 as lipid metabolism-associated molecular signatures of PD and highlights GGPS1-mediated prenylation pathways as a potential mechanistic axis linking metabolic dysregulation to neurodegeneration. These findings provide a framework for future experimental validation and therapeutic exploration targeting lipid-driven pathways in PD. Ongoing studies are evaluating the functional impact of GGPS1 modulation on mitochondrial homeostasis and neuronal vulnerability in human neuronal models.

## Introduction

Parkinson’s disease (PD) is the second most common age-related neurodegenerative disorder and the most prevalent movement disorder (Dorsey et al., 2018). Over the past few decades, the global burden of PD has more than doubled, with an estimated 12 million cases expected by 2040. Clinical symptoms appear only after significant neurodegeneration has taken place, emphasizing the critical need for early diagnosis and effective therapeutics (Scherzer et al., 2007). Understanding the biomolecular mechanisms underlying PD is crucial for developing effective interventions.

PD is characterized by the loss of dopaminergic neurons and pathological aggregation of α-synuclein, a protein that physiologically interacts with lipid membranes and plays a role in the endosome-lysosome system and synaptic signaling, both of which depend heavily on lipid dynamics (Stephens et al., 2019). α-synuclein preferentially binds to negatively charged lipids such as phospholipids and cardiolipin, but excessive binding may promote its aggregation (Galvagnion, 2017, Ryan et al., 2018). Dysregulation of glycosphingolipid metabolism may contribute to neuroinflammation in PD through mechanisms including inflammasome activation, calcium imbalance, blood-brain barrier disruption, immune cell infiltration, and autoantibody production. Alterations in gangliosides (e.g., GM2, GM3) and other lipids such as ceramides, triacylglycerols, sphingomyelins, and phospholipids have been implicated in microglial activation and neurodegeneration (Belarbi et al., 2020, Galper et al., 2022).

Mendelian randomization (MR) is a powerful epidemiological approach using genetic variants as instrumental variables to infer causal relationships between modifiable exposures and disease outcomes. Unlike traditional observational studies, MR reduces confounding and reverse causation, making it valuable to identify potential therapeutic targets. Recent MR studies in PD have revealed causal associations between blood lipids, immune biomarkers, glycemic traits, and disease risk and progression (Chen et al., 2024).

Despite these advances, differentially expressed lipid metabolism-related genes (DE-LMRGs) in PD have not been thoroughly studied. In this study, we apply a comprehensive integrative approach combining transcriptomic analysis, machine learning, immune profiling, and MR to identify and validate lipid metabolism-related biomarkers in PD, evaluate their diagnostic potential, assess genetic causality, and explore potential therapeutic targets. This integrated strategy aims to better understand PD’s biomolecular mechanisms and uncover novel targets for diagnosis and treatment.

## Methods

### Data acquisition and processing

The mRNA expression profiling data for GSE20141 (including 10 PD and 8 control Substantia Nigra pars compacta (SNpc) samples) and GSE20292 (including 18 PD and 11 control SNpc samples) were retrieved from the Gene Expression Omnibus (GEO) database (https://www.ncbi.nlm.nih.gov/geo/). The single-cell dataset GSE184950 included 6 PD tissue samples and 10 control samples on the GPL24676 platform. In total, 776 lipid metabolism-related genes (LMRGs) were obtained from Kyoto Encyclopedia of Genes and Genomes (KEGG) gene set collections on Molecular Signatures Database (MSigDB) (https://www.gsea-msigdb.org/gsea/login.jsp) (Liberzon et al., 2011).

### Weighted gene co-expression network analysis

To further investigate LMRGs in the GSE20141 dataset, the “WGCNA” R package (Langfelder and Horvath, 2008) was used for weighted gene co-expression network analysis (WGCNA). First, all samples (including 10 PD and 8 control SNpc samples from GSE20141) were clustered to detect potential outliers. Second, soft thresholding power (β) based on co-expression similarity was calculated to create the adjacency matrix. Third, genes with similar expression patterns were grouped into modules using hierarchical clustering and dynamic tree cutting, with a minimum module size of 100 genes. The correlation between each module and PD was assessed using Pearson’s correlation. Finally, the module showing the highest correlation with PD was selected for further analysis to identify key module genes.

### Functional analysis of DE-LMRGs

In the GSE20141 dataset, differentially expressed genes (DEGs) between PD and control samples were identified using the “limma” R package (version 3.48.3) with thresholds set at |log_2_FoldChange| > 1 and adjusted p-value < 0.05 (Ritchie et al., 2015). The “ggplot2” R package (version 3.3.5) was used to generate a volcano plot to visualize DEG expression, while the “pheatmap” package (version 1.0.12) illustrated their expression patterns. Differentially expressed lipid metabolism-related genes (DE-LMRGs) were identified by intersecting DEGs, key module genes from WGCNA, and LMRGs from MSigDB using the “ggVennDiagram” package (version 1.2.2) (Gao et al., 2021). Functional enrichment analyses of DE-LMRGs, including Gene Ontology (GO) and Kyoto Encyclopedia of Genes and Genomes (KEGG), were performed using the “clusterProfiler” package (version 4.0.2) (Wu et al., 2021). Results were visualized again using “ggplot2.” Finally, a protein-protein interaction (PPI) network for DE-LMRGs was constructed using the STRING database (https://string-db.org) and visualized with Cytoscape software.

### Identification of biomarkers using machine learning algorithms

To validate the diagnostic potential of DE-LMRGs in PD, their expression was analyzed in both the GSE20141 and GSE20292 datasets using the “ggplot2” R package (version 3.3.5). Candidate genes showing consistent expression patterns and statistically significant differential expression (p < 0.05) in both datasets were selected for further analysis with machine learning methods. Two algorithms were applied to identify PD biomarkers among these candidates: Least Absolute Shrinkage and Selection Operator (LASSO) regression using the “glmnet” R package (version 4.1–4) (Friedman et al., 2010) and the Boruta algorithm (version 7.0.0) (Kursa, 2014), which is a wrapper method built around a random forest classifier. Next, biomarkers for downstream analyses were determined by intersecting the gene sets identified by both LASSO and Boruta.

### Construction and evaluation of a nomogram based on the biomarkers

To further evaluate the diagnostic performance of the identified biomarkers in PD, receiver operating characteristic (ROC) curve analyses were performed using the “pROC” R package (version 1.18.0) (Robin et al., 2011) on the GSE20141 dataset, calculating the area under the curve (AUC). The same analyses were then applied to the GSE20292 dataset, serving as an external validation cohort, to confirm the biomarkers’ generalizability. Additionally, a nomogram incorporating these biomarkers was constructed using the “rms” R package based on the GSE20141 dataset. The nomogram’s predictive accuracy was assessed via calibration curves, decision curve analysis (DCA), and AUC metrics. To explore the biological functions of the biomarkers, Gene Set Enrichment Analysis (GSEA) was conducted using the “clusterProfiler” R package (Yu et al., 2012) with the GSE20141 data, employing the “c5.go.v7.4.entrez.gmt” and “c2.cp.kegg.v7.4.entrez.gmt” gene sets as references. Given the well-established role of SNCA in PD, Spearman correlation analysis was performed to assess associations between biomarkers and SNCA expression. Furthermore, gene-gene interaction networks (GGI) were generated using GeneMANIA (http://www.genemania.org/) to identify genes functionally related to both SNCA and candidate biomarkers.

### Evaluation of immune infiltrating cells and construction of regulatory networks

To assess the immune microenvironment, a single-sample gene set enrichment analysis (ssGSEA) was performed to quantify the relative infiltration levels of 24 immune cell types in PD and control SNpc samples from the GSE20141 dataset. The infiltration abundance was represented by enrichment scores (ES). Correlations between biomarker expression and immune cell infiltration were analyzed using Spearman correlation. To further explore the regulatory mechanisms underlying these biomarkers, TF-mRNA-miRNA and mRNA-miRNA-lncRNA interaction networks were constructed. MicroRNAs (miRNAs) targeting the biomarkers were predicted using the miRDB (https://mirdb.org/) and miRWalk (http://mirwalk.umm.uni-heidelberg.de/) databases, while transcription factors (TFs) regulating the biomarkers were obtained from the TRRUST database (http://www.grnpedia.org/trrust). Subsequently, lncRNAs interacting with the predicted miRNAs were identified through miRTarBase and TarBase databases. Finally, the mRNA–miRNA–lncRNA and TF–mRNA–miRNA regulatory networks were visualized using Cytoscape software (Shannon et al., 2003).

### Design of MR study

To investigate the causal effects of the identified biomarkers on PD, a two-sample MR study was conducted using summary statistics from genome-wide association studies (GWAS). The biomarkers (*LEP*: eqtl-a-ENSG00000174697, sample size: 31,430, SNPs: 17,656; *GGPS1*: eqtl-a-ENSG00000152904, sample size: 31,684, SNPs: 18,426; *DGKD*: eqtl-a-ENSG00000077044, sample size: 31,684, SNPs: 19,506) were considered as exposures, while PD (GWAS ID: ieu-b-7, SNPs: 17,891,936, sample size: 482,730) was the outcome.

Single nucleotide polymorphisms (SNPs) were selected as instrumental variables (IVs) under the following MR assumptions: (i) SNPs are associated with the exposure; (ii) SNPs are not related to confounders affecting both exposure and outcome; (iii) SNPs influence PD risk only through the exposure biomarkers.

Instrumental variables were extracted using the “TwoSampleMR” R package (version 0.5.6) (Hemani et al., 2018). SNPs significantly associated with exposures (p < 5 ×10⁻⁸) were selected, and linkage disequilibrium (LD) pruning was performed by removing SNPs within 10,000 kb and with R² > 0.001.

MR analyses were conducted using five methods: MR-Egger, Weighted median, Inverse variance weighted (IVW), Simple mode, and Weighted mode. The IVW method (for >3 IV SNPs) or Wald ratio method (for ≤3 IV SNPs) was chosen as the primary approach.

Scatter plots visualizing the exposure-outcome relationships were generated based on the IVW results using the ‘mr_scatter_plot’ function. Funnel plots created via the ‘mr_funnel_plot’ function were used to assess the symmetry of causal effects. Forest plots were drawn to evaluate the predictive strength of each SNP for PD risk.

Several sensitivity analyses were performed to assess robustness: Cochran’s Q test evaluated heterogeneity among SNPs; the ‘mr_pleiotropy_test’ function assessed horizontal pleiotropy; and leave-one-out analyses investigated the influence of individual SNPs on overall MR estimates.

Finally, a reverse MR analysis was performed to test potential causal effects of PD on the biomarkers, employing the same methodology.

### Drug prediction and molecular docking

To preliminarily explore potential drugs targeting biomarkers in PD, the Drug–Gene Interaction Database (DGIdb) was used to identify drugs associated with these biomarkers. The chemical structures of key target drugs were obtained from the DrugBank database (https://go.drugbank.com/). Meanwhile, the three-dimensional structures of the biomarkers were downloaded from the RCSB Protein Data Bank (https://www.rcsb.org/). Molecular docking simulations were then performed using AutoDockTools (version 1.5.6) and AutoDock Vina (version 4.2). Finally, PyMOL (version 2.5) was employed to visualize and analyze the interactions and binding affinities between the significant target drugs and key biomarkers.

### Single-cell RNA sequencing data processing

This study was conducted based on the single-cell dataset GSE184950 using the R package Seurat (Version 5.3.0) for data processing and analysis. The quality control criteria were set as follows: cells expressing fewer than 200 genes and genes covered by fewer than 3 cells were filtered out; cells with the number of detected genes greater than 3000 or total gene count greater than 5000 were removed, and the proportion of mitochondrial genes was restricted to below 25%. Subsequently, the NormalizeData function was applied to normalize the data (the expression value of each gene in each cell was divided by the total expression of all genes in that cell, multiplied by 10,000, and log-transformed after adding 1). The FindVariableFeatures function was then used to screen the top 2000 highly variable genes (HVGs) via variance analysis, and the top 10 genes with the highest expression variability were highlighted. Data normalization was performed using the ScaleData function. Principal component analysis (PCA) was conducted on the HVGs using the RunPCA function, and the significance of principal components was evaluated by combining the Jackstraw permutation test (P < 0.05) with the ElbowPlot function. In the clustering analysis stage, unsupervised clustering was performed using the FindNeighbors and FindClusters functions (resolution = 0.5). Cell clusters were annotated according to marker genes reported in the literature. Finally, to clarify the expression profiles of key genes across different cell types, the expression levels of key genes in each cell cluster were visualized, and cells with high expression of key genes were defined as key cells.

## Results

### Identification of nine DE-LMRGs associated with PD

**Fig. 1:**
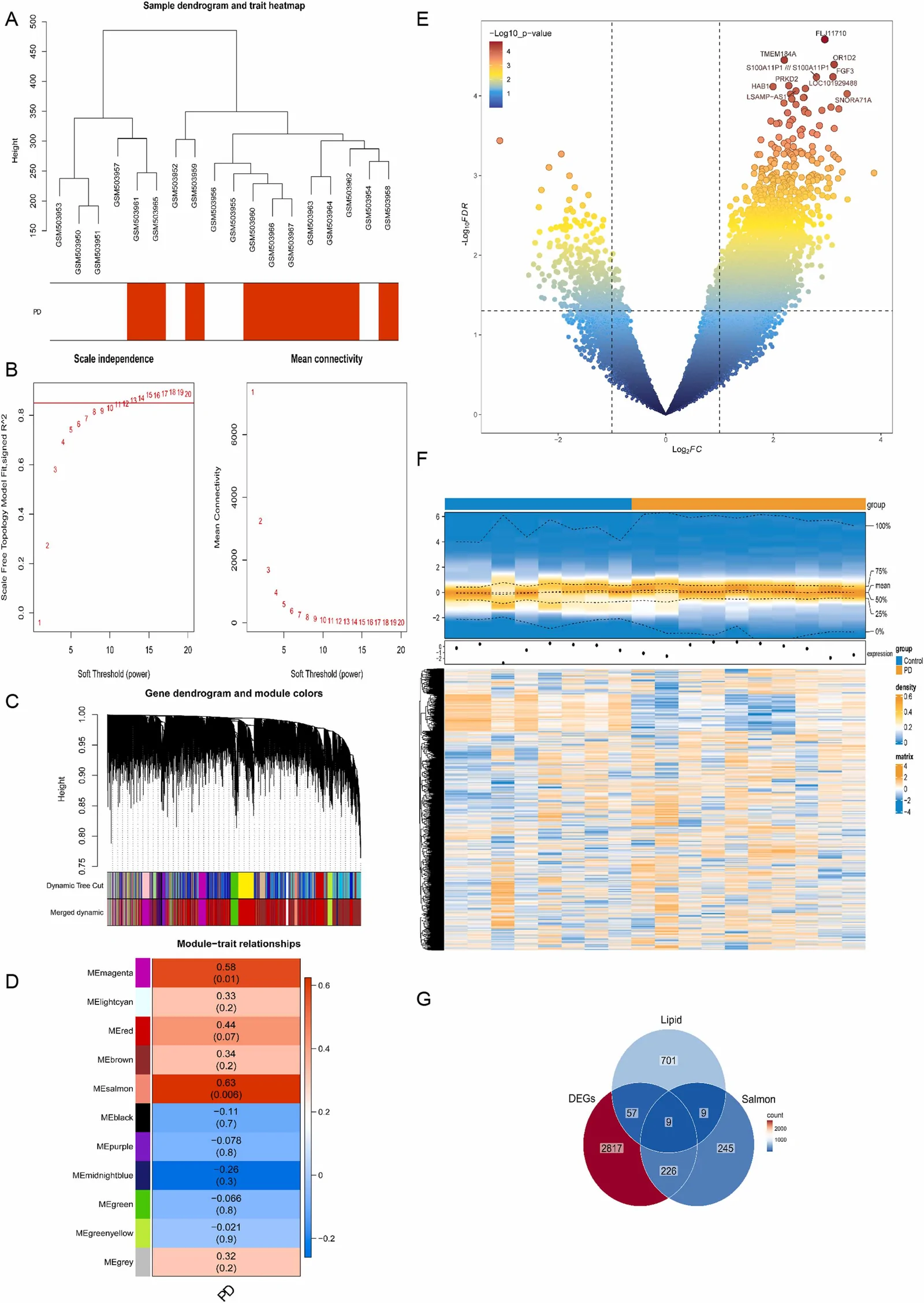
**Identification of lipid metabolism–related genes associated with Parkinson’s disease using WGCNA and differential expression analysis.** (A) Sample clustering dendrogram and trait heatmap for the GSE20141 dataset showing PD and control samples. (B) Determination of the soft-thresholding power used to construct the weighted gene co-expression network. The scale-free topology fit index and mean connectivity were evaluated across different soft-thresholding powers. (C) Gene dendrogram and module color assignment obtained from hierarchical clustering analysis. (D) Module–trait relationship heatmap showing correlations between gene modules and PD status. The salmon module exhibited the strongest correlation with PD. (E) Volcano plot showing differentially expressed genes (DEGs) between PD and control samples. (F) Heatmap illustrating expression patterns of DEGs across samples. (G) Venn diagram showing the intersection between DEGs, lipid metabolism–related genes (LMRGs), and genes in the PD-associated module, identifying nine candidate DE-LMRGs.

As shown in Fig. 1 A, no outlier samples were detected in the GSE20141 dataset. A soft-thresholding power of β = 13 was selected based on the scale-free topology criterion, achieving a scale-free R² = 0.85 (Fig. 1B). Using average hierarchical clustering and dynamic tree cutting, 11 gene co-expression modules were constructed (Fig. 1 C). The correlation between these modules and the PD phenotype is illustrated in Fig. 1D, where the salmon module exhibited the strongest correlation with PD (correlation coefficient = 0.63, *p* = 0.006). A total of 489 genes from this key module were selected for further analysis.

Differential expression analysis using the GSE20141 dataset identified 3109 differentially expressed genes (DEGs) between PD and control samples, including 2703 upregulated and 406 downregulated genes (Figs. 1E and 1F). To refine the identification of PD-related lipid metabolism genes (DE-LMRGs), a Venn diagram analysis was performed by intersecting the salmon module genes (from WGCNA), lipid metabolism-related genes (LMRGs) from the MSigDB database, and DEGs from GSE20141. This integrative approach yielded nine DE-LMRGs: *GGPS1*, *RXRA*, *PLA2G2A*, *LEP*, *LIPH*, *OXCT2*, *CYP4F2*, *MED26*, and *DGKD* (Fig. 1G).

### DE-LMRGs were enriched in the adipocytokine signaling pathway

**Fig. 2:**
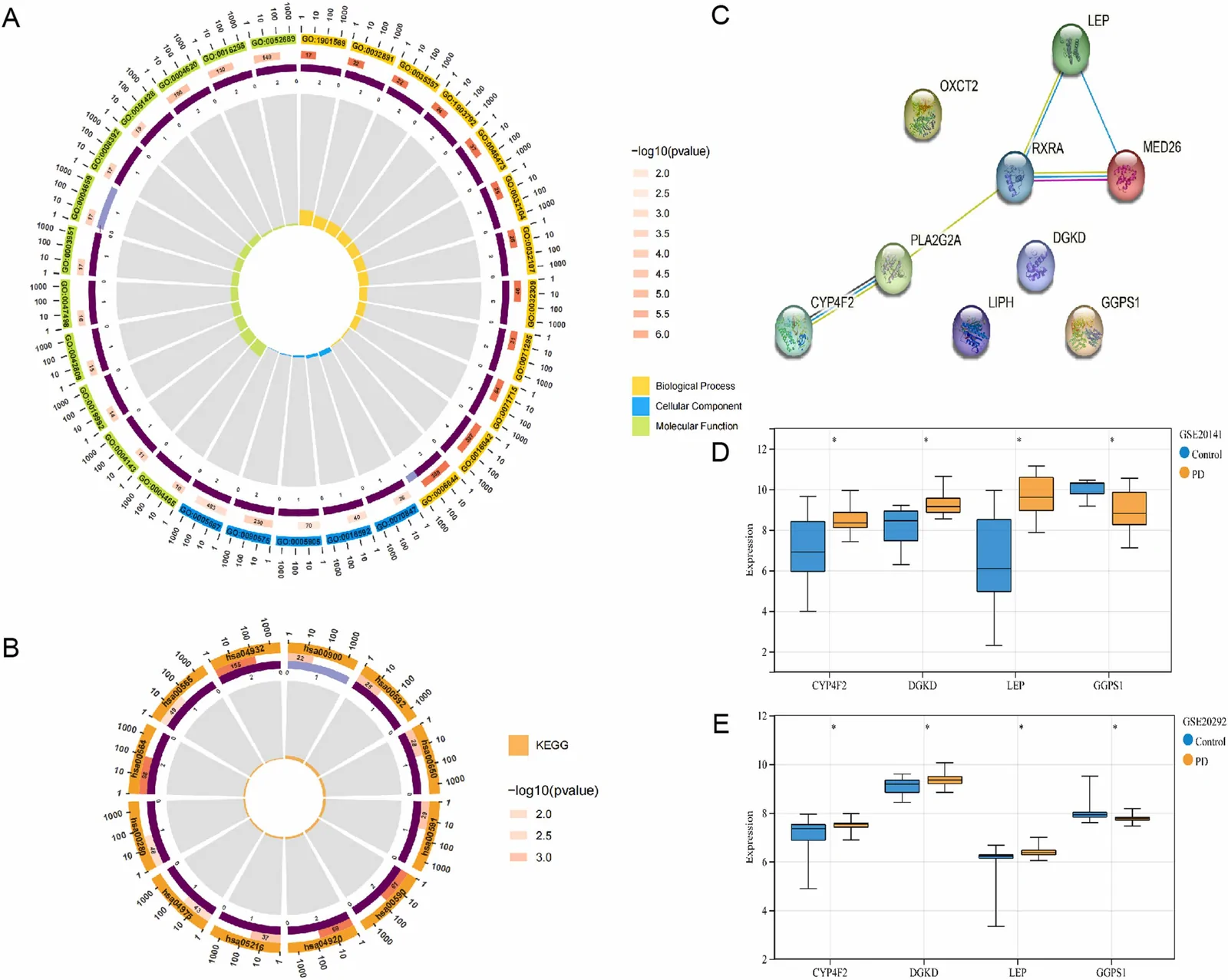
**Functional enrichment analysis and validation of candidate lipid metabolism–related genes.** (A) Gene Ontology (GO) enrichment analysis of the identified DE-LMRGs showing biological process (BP), cellular component (CC), and molecular function (MF) categories. (B) KEGG pathway enrichment analysis indicating pathways associated with lipid metabolism and metabolic signaling. (C) Protein–protein interaction (PPI) network of DE-LMRGs constructed using the STRING database. (D) Expression levels of candidate genes in the GSE20141 dataset comparing PD and control samples. (E) Validation of gene expression patterns in the independent dataset GSE20292.

The GO enrichment analysis revealed that the nine DE-LMRGs were significantly enriched in 341 Gene Ontology (GO) categories (p < 0.05), including 307 biological process (BP), 5 cellular component (CC), and 29 molecular function (MF) terms. As illustrated in Fig. 2A, the BP terms were primarily associated with processes such as the phosphatidic acid metabolic process and the lipid catabolic process. CC terms indicated enrichment in components such as the RNA polymerase II transcription regulator complex and clathrin-coated pit. In terms of MF, DE-LMRGs were mainly involved in phospholipase activity and lipase activity, among others.

KEGG pathway enrichment analysis (Fig. 2B) revealed that DE-LMRGs were involved in 12 pathways, notably including the adipocytokine signaling pathway, glycerophospholipid metabolism, fat digestion and absorption, and ether lipid metabolism. Furthermore, a protein-protein interaction (PPI) network consisting of nine nodes and five edges was constructed (Fig. 2C), in which RXRA showed close interactions with both MED26 and *LEP*.

Finally, of the nine DE-LMRGs, four genes: *GGPS1*, *LEP*, *CYP4F2*, and *DGKD,* demonstrated consistent expression patterns and statistically significant differential expression (p < 0.05) across both the GSE20141 (Fig. 2D) and GSE20292 (Fig. 2E) datasets, supporting their robustness as candidate genes.

### Three genes (*GGPS1*, *LEP*, *DGKD*) were identified as biomarkers for PD

**Fig. 3:**
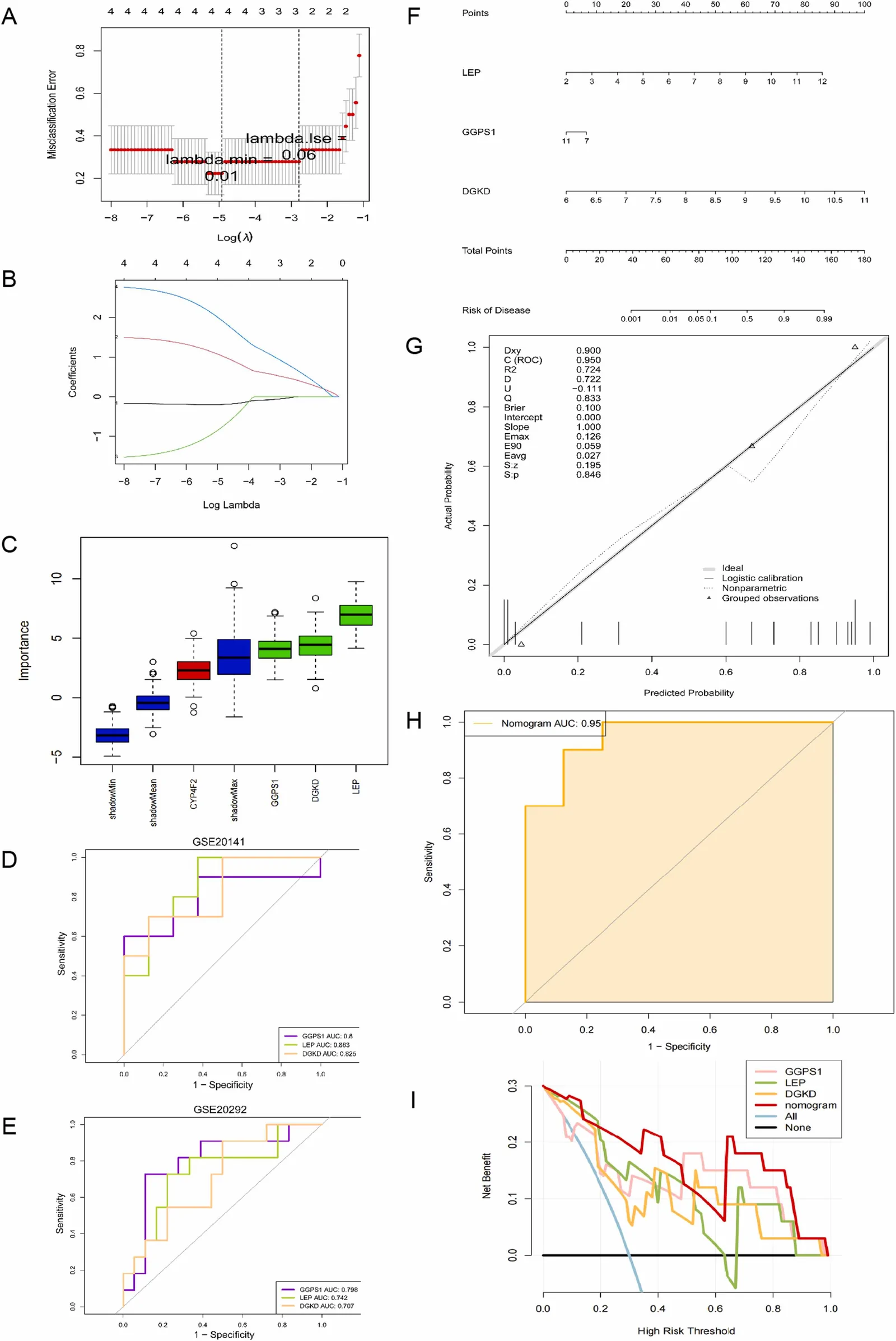
**Identification and validation of diagnostic biomarkers using machine learning models.** (A) Least Absolute Shrinkage and Selection Operator (LASSO) regression analysis used to select candidate biomarkers. (B) LASSO coefficient profiles for genes across different values of the regularization parameter (λ). (C) Boruta feature selection algorithm identifying important genes associated with PD. (D) Receiver operating characteristic (ROC) curves showing the diagnostic performance of the identified biomarkers in the GSE20141 dataset. (E) ROC curves validating biomarker performance in the independent dataset GSE20292. (F) Nomogram constructed using the identified biomarkers to estimate the probability of PD. (G) Calibration curve assessing agreement between predicted and observed PD probabilities. (H) ROC curve of the nomogram model showing its predictive performance. (I) Decision curve analysis evaluating the clinical benefit of the biomarkers and the nomogram model.

Based on the LASSO regression analysis (Figs. 3A and 3B) and the Boruta algorithm (Fig. 3C), three biomarkers: *GGPS1*, *LEP*, and *DGKD,* were identified for subsequent analysis. Notably, ROC curve analyses (Figs. 3D and 3E) demonstrated that these three biomarkers exhibited strong discriminatory power (AUC > 0.7) for distinguishing PD samples from control samples in both the GSE20141 and GSE20292 datasets.

A diagnostic nomogram for PD was constructed based on these three biomarkers (Fig. 3F). The calibration curve (Fig. 3G) and ROC analysis of the nomogram (Fig. 3H) indicated an agreement between predicted and actual outcomes, reflecting excellent predictive performance. Additionally, decision curve analysis (DCA) (Fig. 3I) revealed that the nomogram model offers significant net clinical benefits and supports effective decision-making.

### *GGPS1* was enriched in PD and the neurotrophin signaling pathway

**Fig. 4:**
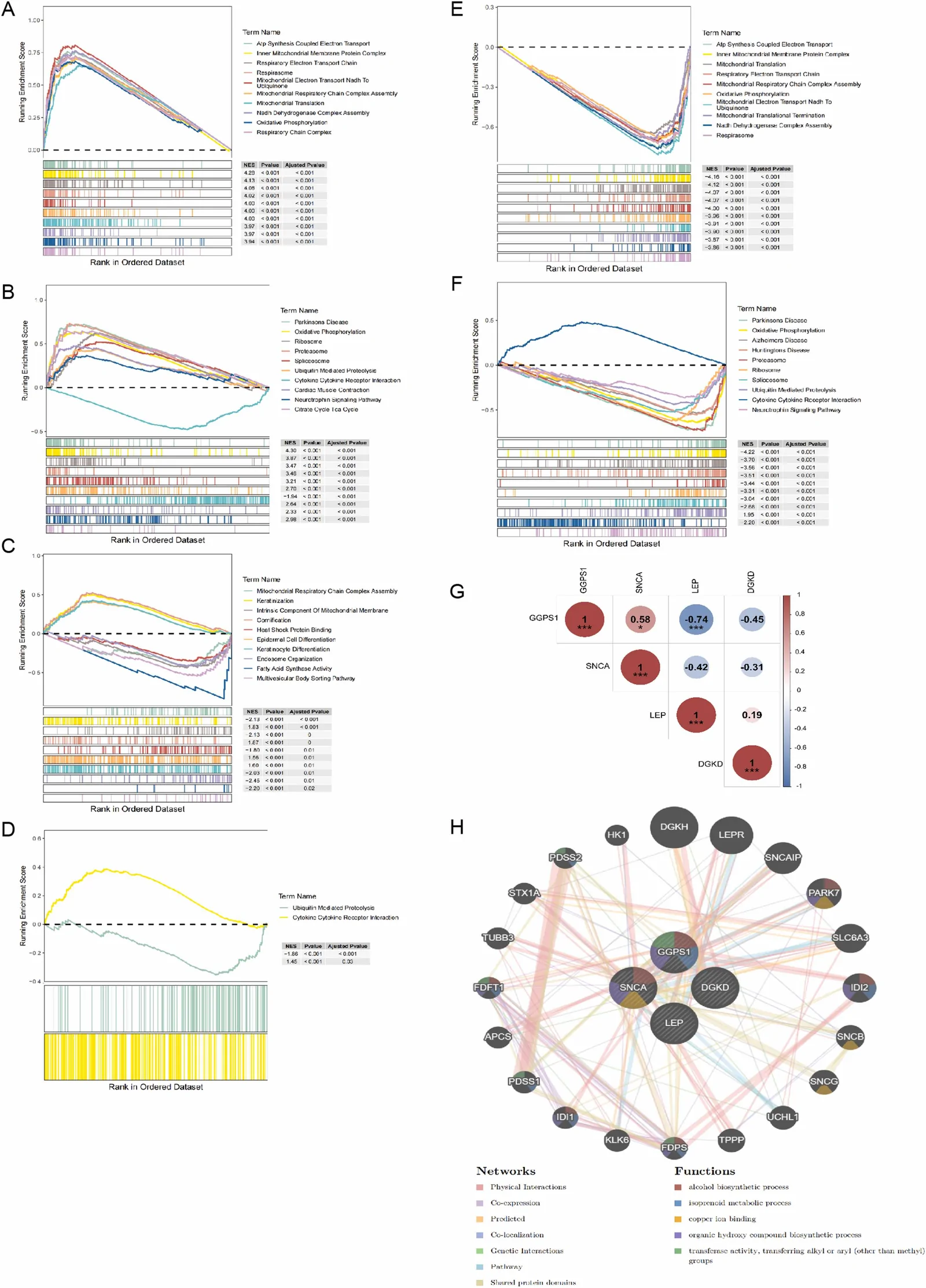
**Functional enrichment and interaction network analyses identify mitochondrial dysfunction and lipid metabolism as major pathways associated with Parkinson's disease.** (A) GSEA demonstrating positively enriched mitochondrial respiratory chain- and oxidative phosphorylation-related GO terms. (B) GSEA of positively enriched KEGG pathways, highlighting Parkinson disease, oxidative phosphorylation, ribosome, proteasome, lysosome, oxidative stress, calcium signaling, and citrate cycle pathways. (C) GSEA illustrating negatively enriched GO biological processes, including mitochondrial respiratory chain complex assembly, inner mitochondrial membrane organization, keratinization, cornification, epithelial cell differentiation, and fatty acid biosynthetic processes. (D) GSEA showing enrichment of ubiquitin-mediated proteolysis and cytokine–cytokine receptor interaction pathways. (E) GSEA demonstrating negatively enriched mitochondrial respiratory chain and oxidative phosphorylation-associated GO terms. (F) GSEA of negatively enriched KEGG pathways, including Parkinson disease, oxidative phosphorylation, Alzheimer's disease, Huntington disease, proteasome, ribosome, spliceosome, lipid metabolism, and cytokine–cytokine receptor interaction. (G) Correlation analysis among the four candidate hub genes (GGPS1, SNCA, LEP, and DGKD). Circle color indicates the direction of correlation (red, positive; blue, negative), circle size reflects correlation strength, and numbers indicate Spearman correlation coefficients. Statistical significance is denoted by asterisks (P < 0.05, P < 0.01, P < 0.001). (H) Gene interaction network of the four hub genes generated using GeneMANIA. Node size reflects network connectivity. Edge colors represent different types of evidence, including physical interactions, co-expression, predicted interactions, co-localization, genetic interactions, pathway associations, and shared protein domains. Functional enrichment analysis indicates that the network is primarily associated with alcohol biosynthetic process, isoprenoid metabolic process, copper ion binding, organic hydroxy compound biosynthetic process, and transferase activity involved in transferring alkyl or aryl groups.

GSEA plots indicated that *GGPS1* was enriched in pathways such as PD, neurotrophin signaling, and mitochondrial translation (Figs. 4A and 4B), while DGKD was enriched in cytokine-cytokine receptor interaction and fatty acid synthase activity (Fig. 4C and 4D). *LEP* was enriched in Alzheimer’s disease, neurotrophin signaling, and mitochondrial translational termination pathways (Figs. 4E and 4F). Additionally, *GGPS1* showed a significant positive correlation with SNCA (cor = 0.58, *p* < 0.05) (Fig. 4G). The GGI network revealed that the biomarkers and SNCA were in the inner layer, while predicted genes were distributed in the outer layer (Fig. 4H). Most of the interactions between *SNCA* and *STX1A*, and between *GGPS1* and *SNCAIP*, were physical interactions. These genes were primarily involved in alcohol biosynthetic processes, isoprenoid metabolism, and copper ion binding.

### *GGPS1* was significantly positively correlated with Tcm

**Fig. 5:**
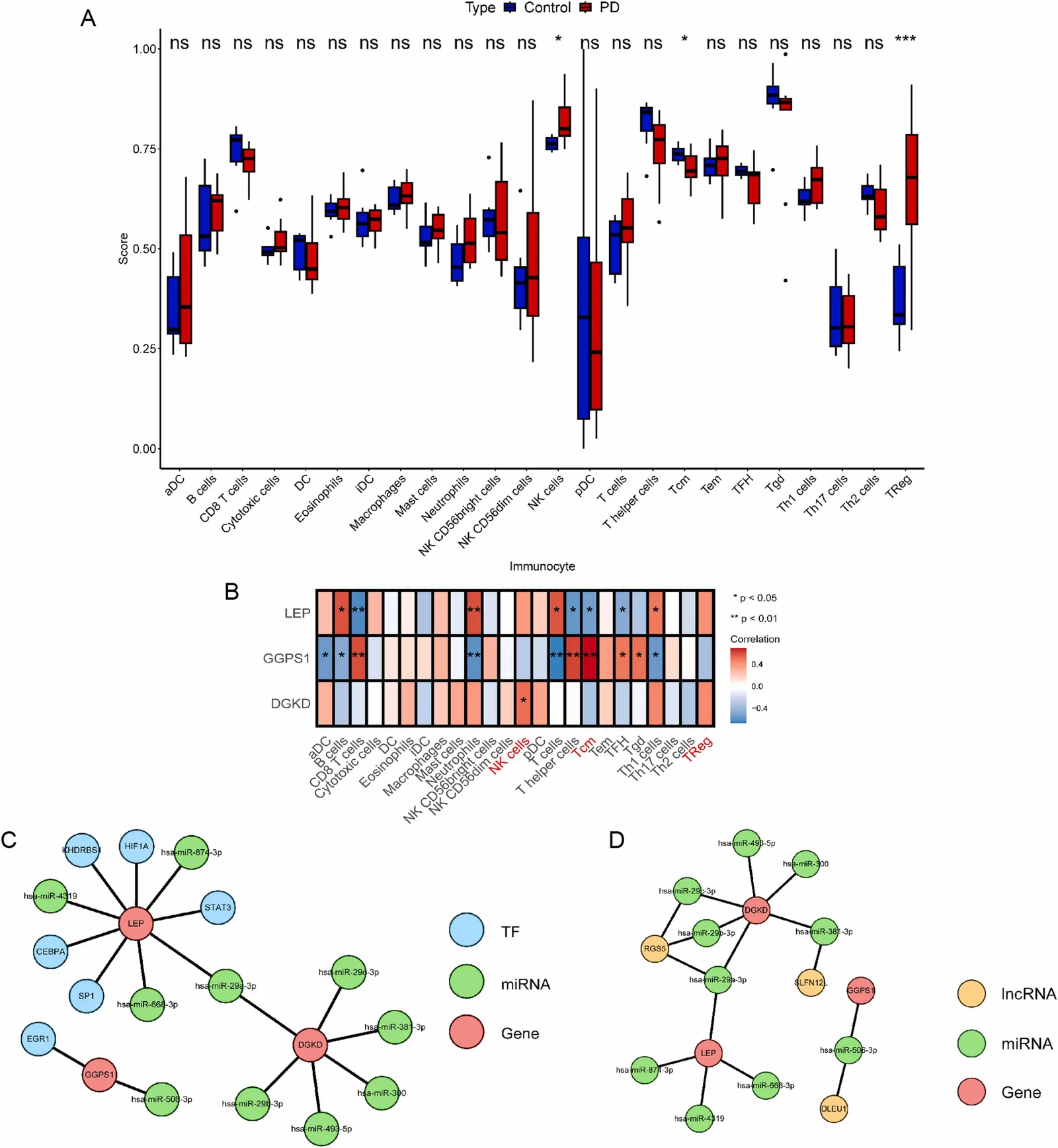
**Immune infiltration analysis and regulatory networks associated with candidate biomarkers.** (A) Comparison of immune cell infiltration levels between PD and control samples using ssGSEA. (B) Heatmap showing correlations between biomarker expression and immune cell infiltration levels. (C) Transcription factor–mRNA–miRNA regulatory network associated with candidate biomarkers. (D) mRNA–miRNA–lncRNA regulatory network illustrating potential regulatory interactions.

As shown in Fig. 5 A, the abundance of three immune cell types: NK cells, Tcm, and Treg differed significantly between PD and control samples in GSE20141 (*p* < 0.05). Correlation analysis between the biomarkers and immune infiltration (Fig. 5B) showed that *LEP* was strongly positively correlated with neutrophils, *GGPS1* with Tcm, and *DGKD* with NK cells. Moreover, the TF–mRNA–miRNA regulatory network (Fig. 5 C) revealed three miRNAs and five TFs targeting *LEP*, one miRNA and one TF regulating *GGPS1*, and six miRNAs regulating *DGKD*. For example, hsa-miR-4319 may regulate *CEBPA* via *LEP*, hsa-miR-506–3p may regulate *EGR1* via *GGPS1*, and hsa-miR-29b-3p may regulate *DGKD*. In addition, the mRNA–miRNA–lncRNA network (Fig. 5D) indicated that RGS5 could regulate *DGKD* through hsa-miR-29b-3p, as well as *LEP* through hsa-miR-29a-3p, while *DLEU1* may regulate *GGPS1* via hsa-miR-506–3p.

### *GGPS1* had a positive effect on PD

**Fig. 6:**
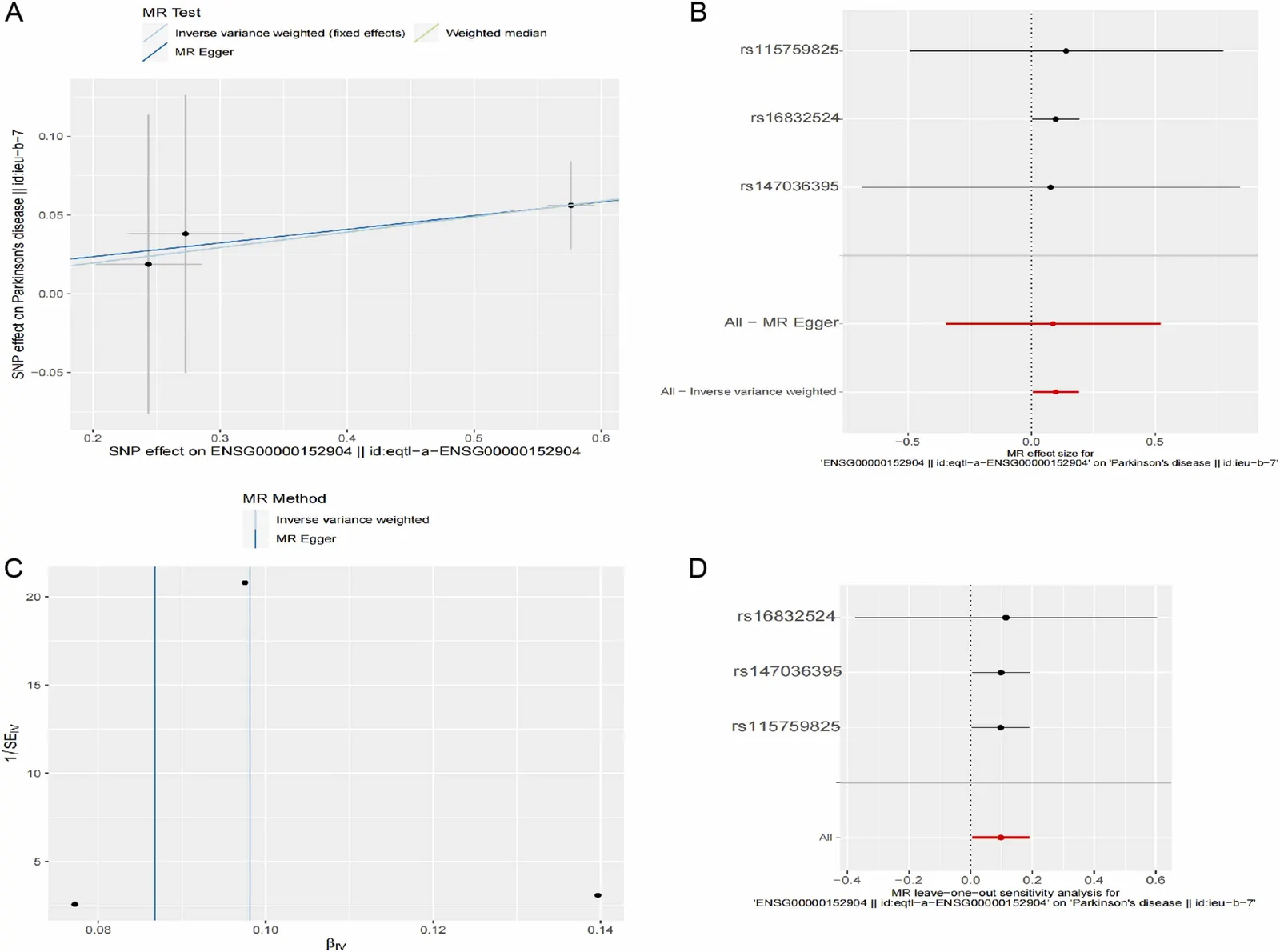
**Mendelian randomization analysis evaluating the causal relationship between biomarkers and Parkinson’s disease.** (A) Scatter plot showing the causal effect estimates of SNPs associated with *GGPS1* on PD risk using different MR methods. (B) Forest plot showing causal effect estimates of individual SNPs and the combined MR effect. (C) Funnel plot assessing heterogeneity and potential pleiotropy among instrumental variables. (D) Leave-one-out sensitivity analysis evaluating the robustness of the MR results.

After SNP screening, two variants were found to be associated with *LEP*, one with *DGKD*, and three with *GGPS1*. Among these, only *GGPS1* was identified as a genetic risk factor for PD in MR analysis (IVW *p* = 0.04, β = 0.10) (Table 1 and Fig. 6A). The IVW results (Fig. 6B) confirmed that the overall causal effect of *GGPS1*-associated SNPs was positive, supporting *GGPS1* as a potential risk factor for PD. However, reverse MR analysis showed no causal effect of PD on *GGPS1* (IVW *p* = 0.54) (Table S1). The funnel plot showed a relatively uniform and symmetrical distribution of SNPs in MR analysis (Fig. 6C). Sensitivity analyses confirmed the absence of heterogeneity (*p* for Cochran’s Q MR-Egger = 0.90) and horizontal pleiotropy (*p* for MR-Egger intercept = 0.97) (Table 2). The leave-one-out analysis further validated the robustness of the MR results, as the exclusion of individual SNPs did not significantly alter the outcome (Fig. 6D).

**Table 1:** Mendelian randomization estimates for gene expression and Parkinson’s disease. nsnp: number of SNP instruments; b: effect estimate; se: standard error; IVW: inverse variance weighted.

**Exposure**	**Exposure ID**	**Method**	**nsnp**	**b**	**se**	**pval**	**Outcome**
*DGKD*	eqtl-a-ENSG00000077044	Wald ratio	1	-0.08	0.10	0.43	Parkinson's disease || ID: ieu-b-7
*GGPS1*	eqtl-a-ENSG00000152904	MR Egger	3	0.09	0.22	0.76	
		Weighted median	3	0.10	0.05	0.04	
		Inverse variance weighted	3	0.10	0.05	0.04	
		Simple mode	3	0.09	0.11	0.51	
		Weighted mode	3	0.10	0.05	0.19	
*LEP*	eqtl-a-ENSG00000174697	Wald ratio	1	0.15	0.19	0.45

**Table 2:** Heterogeneity and pleiotropy tests for Mendelian randomization analysis. Q: Cochran’s Q statistic; Q_df: degrees of freedom; Q_pval: heterogeneity P value; se: standard error.

		**Heterogeneity**				**Horizontal Pleiotropy**		
**Exposure ID**	**Outcome**	**Method**	**Q**	**Q_df**	**Q_pval**	**egger_intercept**	**se**	**pval**
*GGPS1*/eqtl-a-ENSG00000152904	Parkinson's disease || ID:ieu-b-7	MR Egger	0.02	1	0.90	0.01	0.12	0.97
		Inverse variance weighted	0.02	2	0.99	N/A	N/A	N/A

### Potential drugs for biomarkers in PD

**Fig. 7:**
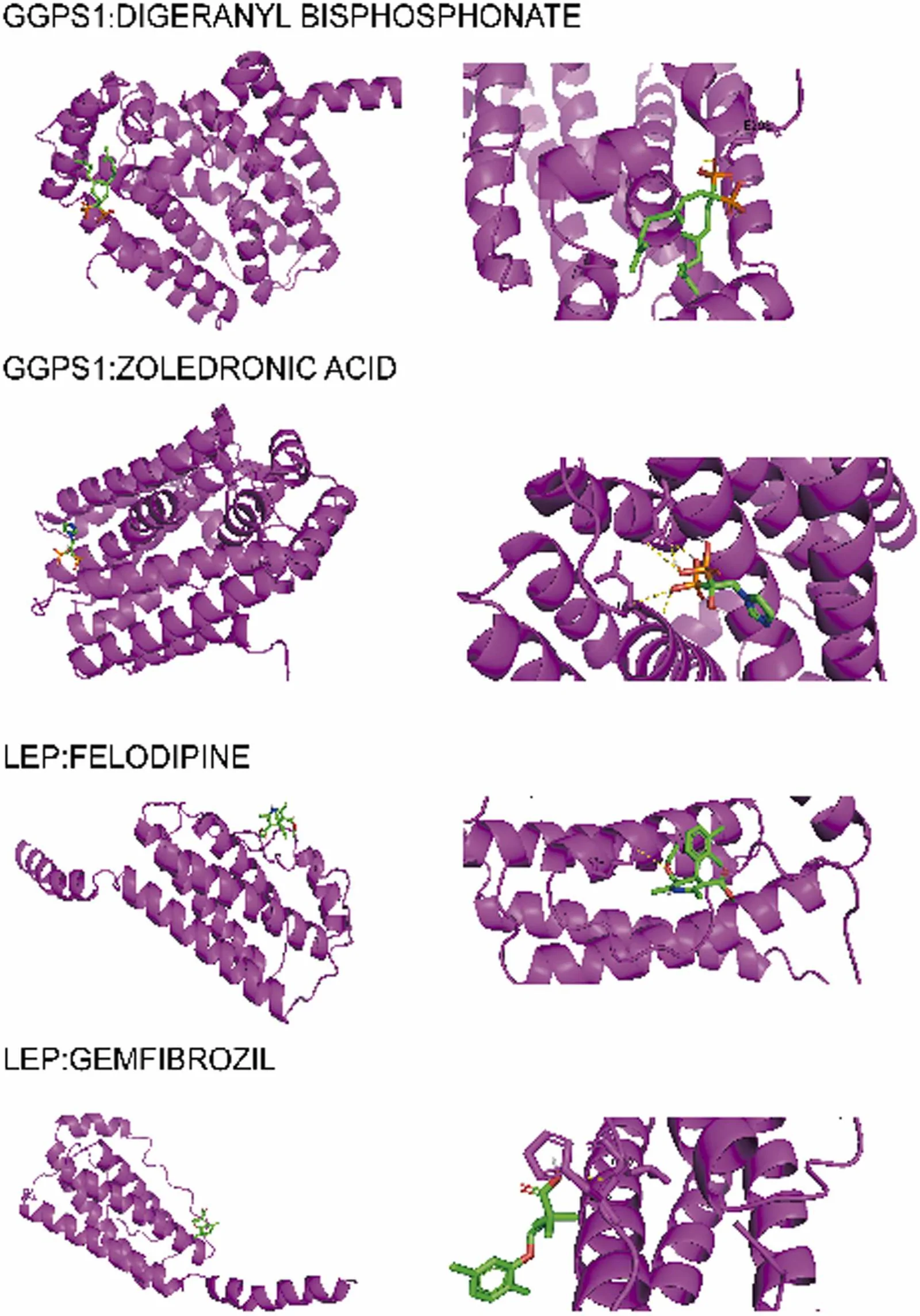
**Molecular docking analysis of candidate drugs targeting identified biomarkers.** Structural docking models illustrating interactions between candidate drugs and biomarker proteins. *GGPS1* interactions with digeranyl bisphosphonate and zoledronic acid are shown in the upper panels, while *LEP* interactions with felodipine and gemfibrozil are shown in the lower panels. Binding poses highlight potential interaction sites within the protein structures.

To explore potential therapeutic targets, drug–gene interactions were queried using the DGIdb database (Table 3). Two candidate drugs were identified for *GGPS1*—Digeranyl bisphosphonate and Zoledronic acid—and 15 drugs were found to be associated with *LEP*, including Methimazole, Felodipine, and Troglitazone. Subsequently, molecular docking was performed using AutoDock (Fig. 7). The binding energies between *GGPS1* and Digeranyl bisphosphonate and Zoledronic acid were −4.4 kcal/mol and −4.3 kcal/mol, respectively. Meanwhile, the docking energies between *LEP* and Gemfibrozil and Felodipine were −5.2 kcal/mol and −4.3 kcal/mol, respectively, indicating favorable binding affinities.

**Table 3:** Drug–gene interactions identified from public databases. DTC: Drug–Target Interaction database, TdgClinicalTrial: Therapeutic Target Database; NCI: National Cancer Institute; PharmGKB: Pharmacogenomics Knowledgebase.

**GENE**	**Drug**	**Sources**
*GGPS1*	DIGERANYLBISPHOSPHONATE	DTC
*GGPS1*	ZOLEDRONIC ACID	TdgClinicalTrial
*LEP*	MIRTAZAPINE	NCI
*LEP*	METHIMAZOLE	NCI
*LEP*	BROMOCRIPTINE	NCI
*LEP*	LOVASTATIN	NCI
*LEP*	METHADONE	NCI
*LEP*	PROPYLTHIOURACIL	NCI
*LEP*	CARVEDILOL	NCI
*LEP*	RISPERIDONE	NCI|PharmGKB
*LEP*	GEMFIBROZIL	NCI
*LEP*	LAMIVUDINE	NCI
*LEP*	FELODIPINE	NCI
*LEP*	CLOZAPINE	NCI
*LEP*	TROGLITAZONE	NCI
*LEP*	SOYBEAN	OIL
*LEP*	OLANZAPINE	PharmGKB

### Single-cell landscape of lipid metabolism genes in PD

**Fig. 8:**
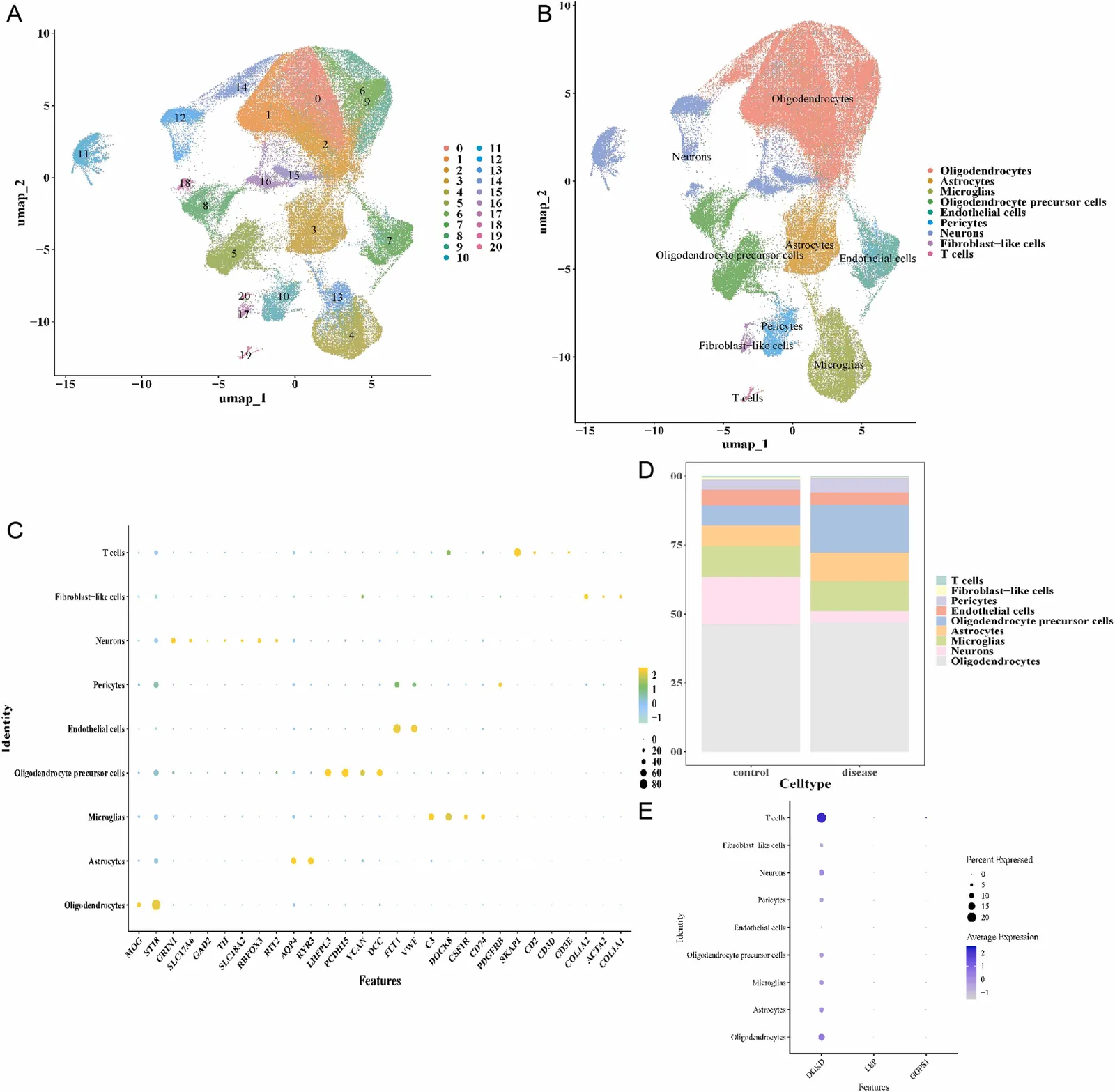
**Identification of PD cell types and characterization of marker gene expression.** (A-B) UMAP dimensionality reduction plots showed the clustering distribution of single cells from brain tissue. Each dot represented an individual cell, with different colors indicating distinct cell types or subpopulations. The horizontal and vertical axes (UMAP_1 and UMAP_2) represented the two-dimensional projection after dimensionality reduction. (C) Dot plots displayed the expression of representative marker genes across different cell clusters. Dot size indicated the proportion of cells expressing the gene, and color intensity reflected the expression level. (D) Bar plot showed the proportion of each cell type within the total cell population. The vertical axis represented the proportion value (ranging from 0 to 1.00). (E) Bubble plot illustrated the expression profiles of S-biomarkers across various cell clusters. Color scale represented the average expression level (ranging from –1 to 2), and dot size reflected the proportion of cells expressing the gene within the corresponding cluster.

After completing the whole transcriptome sequencing analysis, we further performed single-cell RNA sequencing analysis based on the GSE184950 dataset to explore the potential pathogenesis of PD at the cellular level. First, we visualized the nFeature_RNA and nCount_RNA of PD samples before and after quality control. Prior to quality control, the dataset contained 162,172 cells and 26,723 genes, whereas after quality control, 116,105 cells and 26,723 genes were retained (Supplementary Figure 1A-B). After data normalization, we screened 2000 HVGs for subsequent analysis and identified the top 10 HVGs with the highest variability (such as GALNTL6, COL1A1, and CEMIP) (Supplementary Figure 1 C). Following PCA, we selected the top 30 PCs for downstream analysis (P < 0.05) (Supplementary Figure 1D-E). Unsupervised clustering analysis revealed that PD-associated cells formed 21 distinct cell clusters (Fig. 8A). These cell clusters were annotated into nine different cell types based on marker genes (Fig. 8B), including oligodendrocytes, neurons, astrocytes, oligodendrocyte precursor cells, endothelial cells, microglia, pericytes, T cells, and fibroblast-like cells. A bubble plot further verified the accuracy of cell annotation and clearly demonstrated the high specificity of marker gene expression in each cell type (Fig. 8C). The proportion analysis of cell types revealed the distribution of different cell populations across samples (Fig. 8D). Analysis of key gene expression showed that DGKD exhibited the highest expression level in T cells among all single cells. Therefore, T cells were defined as the key cell type for subsequent analyses (Fig. 8E).

## DISCUSSION

PD is a complex neurodegenerative disorder characterized by the progressive loss of dopaminergic neurons and accumulation of α-synuclein aggregates. Emerging evidence implicates dysregulation of lipid metabolism as a significant contributor to PD pathogenesis. Lipids are fundamental components of neuronal membranes and are involved in signaling, energy homeostasis, and vesicle trafficking processes essential for neuronal survival and function (Zhang et al., 2024, Tracey et al., 2018). Lipid metabolism, at the intersection of neuronal energy balance and immune regulation, has therefore emerged as a promising but underexplored contributor to PD pathology.

To understand the evolution of this research area, it is instructive to examine recent advances. Over the past decade, studies have linked lipid pathways, including the mevalonate-prenylation route, glycerolipid and fatty-acid metabolism, and cholesterol/ceramide homeostasis to neuronal vulnerability in PD (Galper et al., 2022, Alecu and Bennett, 2019, Nuber et al., 2025, Barbuti et al., 2025). While many transcriptomic reports treated lipid alterations as downstream correlations of neurodegeneration, recent integrative and genetic approaches have begun to suggest upstream contributions of lipid dysregulation to PD risk (Cooper et al., 2024). To test this hypothesis and identify actionable biomolecular targets, we employed an integrative, multi-layer analytical framework to identify key lipid metabolism-related biomarkers in PD.

Our approach combined WGCNA, differential gene expression profiling, machine-learning-based prioritization, MR, and drug–gene interaction prediction. MR reduces confounding and reverse causation, whereas WGCNA captures gene–gene associations beyond differential expression. By intersecting these independent analytical layers, our study bridges statistical association with mechanistic inference and establishes a reproducible framework for exploring causal biomolecular axes in PD. Through this pipeline, we identified *GGPS1*, *LEP*, and *DGKD* as robust candidate biomarkers with diagnostic potential. Although these genes act through distinct mechanisms, they converge in a shared lipid–immune regulatory network that influences neuronal survival and stress response.

*GGPS1*, encoding geranylgeranyl diphosphate synthase 1, catalyzes the synthesis of GGPP, a crucial substrate for prenylation of small GTPases such as Rab and Rho families. These GTPases regulate intracellular vesicle trafficking, mitochondrial dynamics, and autophagy, all processes impaired in PD (Yamano et al., 2018, Wauters et al., 2020, Steger et al., 2016). The causal link of *GGPS1* to PD identified via MR supports the hypothesis that defects in protein prenylation contribute to dopaminergic neuronal vulnerability. Such disturbances may disrupt vesicular recycling and synaptic energy metabolism and promote selective neuronal stress. This aligns with preclinical evidence that modulation of the mevalonate pathway impacts neurodegenerative processes (Bi et al., 2004).

*LEP* (leptin), a hormone central to energy homeostasis and lipid metabolism, also emerged as a significant biomarker, though MR did not support a causal relationship. Leptin exerts neuroprotective effects by enhancing mitochondrial function and preventing ATP depletion, potentially mitigating PD progression (Cheng et al., 2020, Ho et al., 2010). Similarly, *DGKD* (diacylglycerol kinase delta), involved in lipid signaling pathways regulating protein kinase C and mTOR (Liu et al., 2024), was strongly associated with PD but lacked causal evidence in MR. *DGKD*-related pathways may influence α-synuclein aggregation and mitochondrial health, requiring further exploration.

Our gene set enrichment analyses confirmed that lipid metabolic pathways such as glycerolipid metabolism, fatty acid elongation, and PPAR signaling are significantly enriched in PD, supporting previous transcriptomic findings (Galper et al., 2022, Hällqvist et al., 2025, Yazar et al., 2021). These results align with prior evidence showing that lipid metabolism genes are enriched in PD-affected brain regions and influence endoplasmic reticulum stress, lipid droplet accumulation, and vesicular dysfunction (Xicoy et al., 2020, Nido et al., 2020). Importantly, our findings extend this knowledge by integrating genetic causality through MR, providing a directional framework for interpreting lipid alterations as potential upstream drivers rather than secondary consequences of neuronal loss. In addition to these core lipid pathways, our GSEA also highlighted enrichment in Parkinson's disease-related KEGG and neurotrophin signaling pathways, suggesting that lipid dysregulation intersects with neuronal survival signaling cascades. This pattern supports the idea that lipid metabolism is not isolated from PD pathology but dynamically integrated with cellular stress and energy regulation processes. Given that these metabolic pathways operate within a complex cellular environment, we further examined immune cell composition to understand the cellular context of these biomolecular changes.

Immune cell infiltration analyses revealed dysregulation in natural killer cells, central memory T cells, and regulatory T cells, suggesting an interplay between lipid metabolism and neuroinflammation in PD pathogenesis. These immune changes are particularly relevant because T-cell activation relies on lipid-driven energy remodeling (Lim et al., 2022). The correlation between *GGPS1* and Tcm abundance therefore supports the presence of a lipid-immune feedback loop that may sustain chronic inflammation in PD (Tiwari and Simons, 2025). Specifically, NK cells contribute to cytotoxicity and may accelerate neuronal stress via inflammatory cytokine release. Tcm cells maintain long-term immune memory and can perpetuate low-grade inflammation, while Treg cells normally restrain excessive immune responses, and their dysfunction may allow chronic neuroinflammatory signaling. These shifts together support an immune-metabolic imbalance that links peripheral immune activation to central neurodegeneration in PD (Tansey et al., 2022).

Receiver operating characteristic analyses demonstrated that *GGPS1*, *LEP*, and *DGKD* provide strong diagnostic performance, and their combination in a nomogram model further improved accuracy (AUC = 0.832). This AUC value indicates a discriminatory power between PD and control samples, and cross-validation using GSE20292 further supports model generalizability. These findings suggest that multi-marker panels integrating lipid metabolism genes could enhance early diagnosis and patient stratification in PD.

Drug repurposing analyses prioritized zoledronic acid and felodipine as candidate therapeutics targeting lipid metabolism-related pathways. Zoledronic acid, a nitrogen-containing bisphosphonate, inhibits the mevalonate pathway by blocking farnesyl pyrophosphate synthase, thereby reducing excessive protein prenylation and downstream neuroinflammatory signaling (Xie et al., 2015, Rietkötter et al., 2013). Through this mechanism, it may help restore endolysosomal and mitochondrial homeostasis, two processes consistently impaired in PD. Felodipine, a dihydropyridine calcium channel blocker, activates autophagy via the Ca²⁺–AMPK–TFEB signaling axis and promotes the clearance of α-synuclein aggregates, leading to reduced neuronal toxicity in experimental PD models (Siddiqi et al., 2019, Gao et al., 2019). These findings warrant experimental validation to confirm efficacy and mechanisms.

Notably, felodipine induces brain-penetrant autophagy induction and epidemiological evidence suggests reduced PD incidence among hypertensive patients treated with this agent (Siddiqi et al., 2019). In parallel, zoledronic acid is currently under evaluation in the TOPAZ randomized trial in PD populations, offering a practical route to assess its neuroprotective and systemic benefits (https://clinicaltrials.gov/study/NCT03924414). Both compounds converge on the metabolic–autophagic interface identified in our analyses, supporting lipid modulation as a promising disease-modifying strategy.

Based on the systematic analysis of the GSE184950 dataset, we successfully constructed a single-cell transcriptomic atlas of the substantia nigra region, obtaining 116,105 high-quality cells after quality control and annotating them into 9 major cell types. This cellular composition profile was largely consistent with previously published single-cell studies of the substantia nigra, suggesting that our data quality and annotation strategy were reliable. Notably, the proportional distribution of each cell type differed markedly across PD-related samples, indicating that the cellular microenvironment of the substantia nigra might have undergone remodeling under disease conditions, which was consistent with the pathological features of neuroinflammation and glial activation in PD. These shifts in cell type proportions likely reflected disease-associated cell death, reactive gliosis, or immune cell infiltration. Among these, we found that DGKD exhibited the highest expression level in T cells, a finding that warrants further attention. The role of T cell infiltration in PD pathogenesis has been increasingly substantiated, with peripheral T cells gaining entry into the brain parenchyma through a compromised blood-brain barrier, where they released inflammatory cytokines and contributed to dopaminergic neuron damage. As a member of the diacylglycerol kinase family, DGKD is involved in phosphatidylinositol signaling and lipid metabolism regulation. Its elevated expression in T cells suggested that lipid metabolic reprogramming might participate in modulating T cell activation status and functional polarization, thereby influencing neuroimmune inflammatory responses in PD. Meanwhile, the key genes GGPS1 and LEP were also detected at varying expression levels across other cell types, implying that these genes might synergistically contribute to PD pathology through multiple cell types. Overall, our single-cell analysis provided a novel perspective for understanding the link between lipid metabolism dysregulation and PD pathogenesis and offered potential targets for future cell type-specific intervention strategies. However, it should be noted that these results were solely descriptive at the expression level, and the causal relationship between DGKD overexpression in T cells and PD pathogenesis, as well as the intercellular communication networks mediated by lipid metabolic signals between T cells and other cell types, remain to be further validated through functional experiments and cell-cell interaction analyses.

Limitations of our study include the use of publicly available datasets with limited sample sizes that may not fully capture brain-specific regulatory effects, potential postmortem bias in transcriptomic profiles, and possible pleiotropic effects in the MR analysis. Specifically, the discovery datasets (GSE20141 and GSE20292) have relatively small sample sizes, and post-mortem brain tissue profiles can be highly susceptible to confounding factors such as post-mortem interval, age, and cause of death. While age and sex were found to be balanced between groups in GSE20292, these and other potential confounders were not fully adjustable due to data availability, which may affect the robustness of the diagnostic model. In addition, the absence of additional independent validation datasets and the lack of in vitro experimental confirmation may limit the generalizability of our findings. Future studies incorporating longitudinal designs and multi-cohort validation, as well as single-cell RNA-seq and metabolomics integration, could further clarify lipid-immune interactions and strengthen the mechanistic understanding of *GGPS1* and related biomarkers in PD. Despite these limitations, our study provides a comprehensive framework for investigating lipid metabolism in PD. Overall, our findings suggest that dysregulation of lipid metabolism driven by key genes such as *GGPS1*, *LEP*, and *DGKD* may disturb mitochondrial function and immune homeostasis, thereby contributing to PD pathogenesis.

## CRediT authorship contribution statement

**Dunhui Li:** Writing – original draft, Supervision, Funding acquisition, Data curation, Conceptualization. **Suxiang Chen:** Writing – original draft, Visualization, Supervision, Formal analysis. **Tao Wang:** Writing – original draft, Supervision, Funding acquisition, Formal analysis, Data curation, Conceptualization. **Calikusu Fatma:** Writing – review & editing, Writing – original draft, Project administration, Investigation.

## Declaration of Competing Interest

The authors declare that they have no known competing financial interests or personal relationships that could have appeared to influence the work reported in this paper.
